# A case of primary mesenteric synovial sarcoma: a challenging presentation

**DOI:** 10.1186/s40792-023-01744-2

**Published:** 2023-09-06

**Authors:** Nihed Abdessayed, Malek Barka, Samiha Mabrouk, Zeineb Nfikha, Zeineb Maatoug, Yosra Fejji, Mohamed Salah Jarrar, Sabri Youssef, Moncef Mokni

**Affiliations:** 1 Department of Pathology, Farhat Hached University Hospital, Sousse, Tunisia; 2 Department of General and Digestive Surgery, Faculty of Medicine of Sousse, Farhat Hached University Hospital, Sousse, Tunisia; 3 Research Lab: Transfer in Technology in Anatomic Pathology (LR12SP08), Sousse, Tunisia

**Keywords:** Synovial, Sarcoma, Mesenteric, Intra-abdominal, Primary

## Abstract

**Background:**

Synovial sarcoma is an uncommon soft tissue malignancy that mainly occurs near tendon sheath and bone joints. Primary intra-abdominal location is exceedingly rare and characterized by non-specific clinical signs.

**Case presentation:**

We report the case of a young female without medical history who presented with acute abdominopelvic pain. Ultrasound echography revealed a right mass measuring 7 cm in greater diameter cystic with solid areas, likely of ovarian origin. A coelioscopy with peritoneal biopsies was performed. Histological examination with immunohistochemistry concluded the diagnosis of GIST. The patient was referred to the surgery department and after laboratory routine analysis and computed tomography, the patient was proposed to surgical management. Per-operative findings revealed a mesenteric mass locally invading the greater omentum and the appendicular wall. Pathological examination with immunochemistry confirmed the diagnosis of mesenteric monophasic synovial sarcoma invading the appendicular wall with positive surgical margins. Chemotherapy was proposed with a good response. Our patient is free from disease 9 months later.

**Conclusions:**

We aimed through this case report to discuss mesenteric presentation monophasic SS, mimicking ovarian malignancy, emphasizing clinicopathological features and differential diagnoses.

## Background

Synovial sarcoma (SS) is a rare and aggressive tumor, that represents up to 10% of all soft tissue malignancies [1, 2]. In contrast to its name, SS is not related to synovial tissue and it is considered of tumor unknown origin [3]. The disease occurs mainly near tendon sheath and bone joints. Other locations such as head and neck or retroperitoneum are described too [4]. Primary intra-abdominal SS is exceedingly rare, with only a few cases reported worldwide. Clinical presentation is non-specific and symptoms vary behalf on the tumor site [5].

Here, we present a case of primary mesenteric monophasic SS, mimicking ovarian malignancy. Clinico-pathological features and differential diagnoses of this entity will be discussed.

## Case presentation

A 40-year-old female without past medical history presented to the gynecology department for acute abdominopelvic pain. Ultrasound echography revealed a right mass measuring 7 cm in greater diameter cystic with solid areas. A coelioscopy with peritoneal biopsies was performed. Histological examination of these samples showed a spindle cell proliferation with a dense vascular network. Nuclear atypia were scants and some mitotic figures were noticed. At immunohistochemistry, tumor cells showed focal positive staining with SMA, EMA, Cytokeratin AE⁄AE3, and DOG1. C-KIT, CD34, S100, Melan A, PAX8, p53, WT1, calretinin, mesothelin, and D2–40 were negative. The diagnosis of Gastrointestinal stromal tumor (GIST) was retained and the patient was referred to the surgery department for surgical management.

**Fig. 1:**
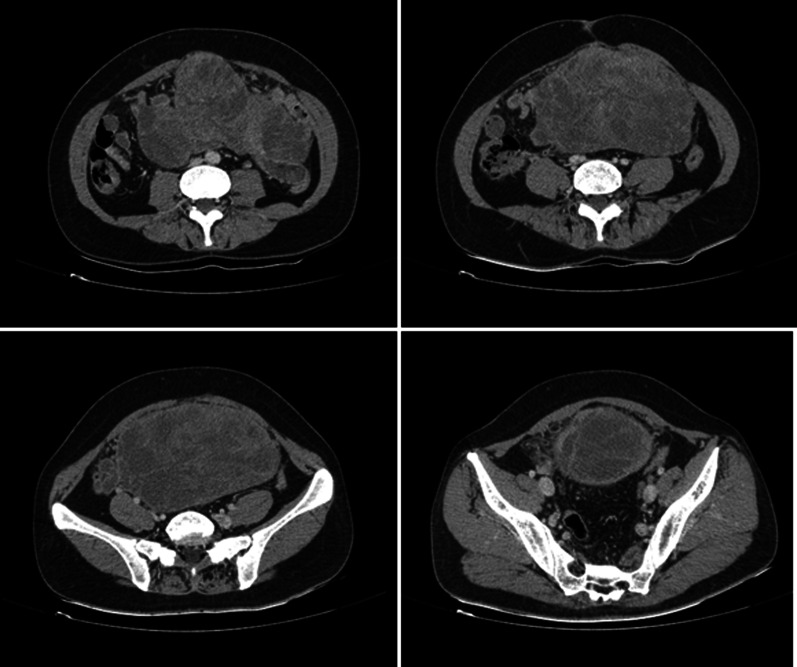
Axial contrast-enhanced computed tomography (CT) of a primary intra-abdominal synovial sarcoma in a 40-year-old woman. A large heterodense, multilobulated lesion with a size of 20 × 15 × 10 cm

A contrast-enhanced computed tomography (CT) (Fig. 1) was performed which showed a large heterodyne, multilobulated lesion sized 20 × 15 × 10 cm occupying the entire pelvis and extending up to the supra-umbilical region.

The right ureter and kidney were closely abutting the lesion, but there was no evidence of locoregional invasion into the adjacent viscera. There was no vascular invasion. Despite all these findings, the lesion was deemed resectable.

**Fig. 2:**
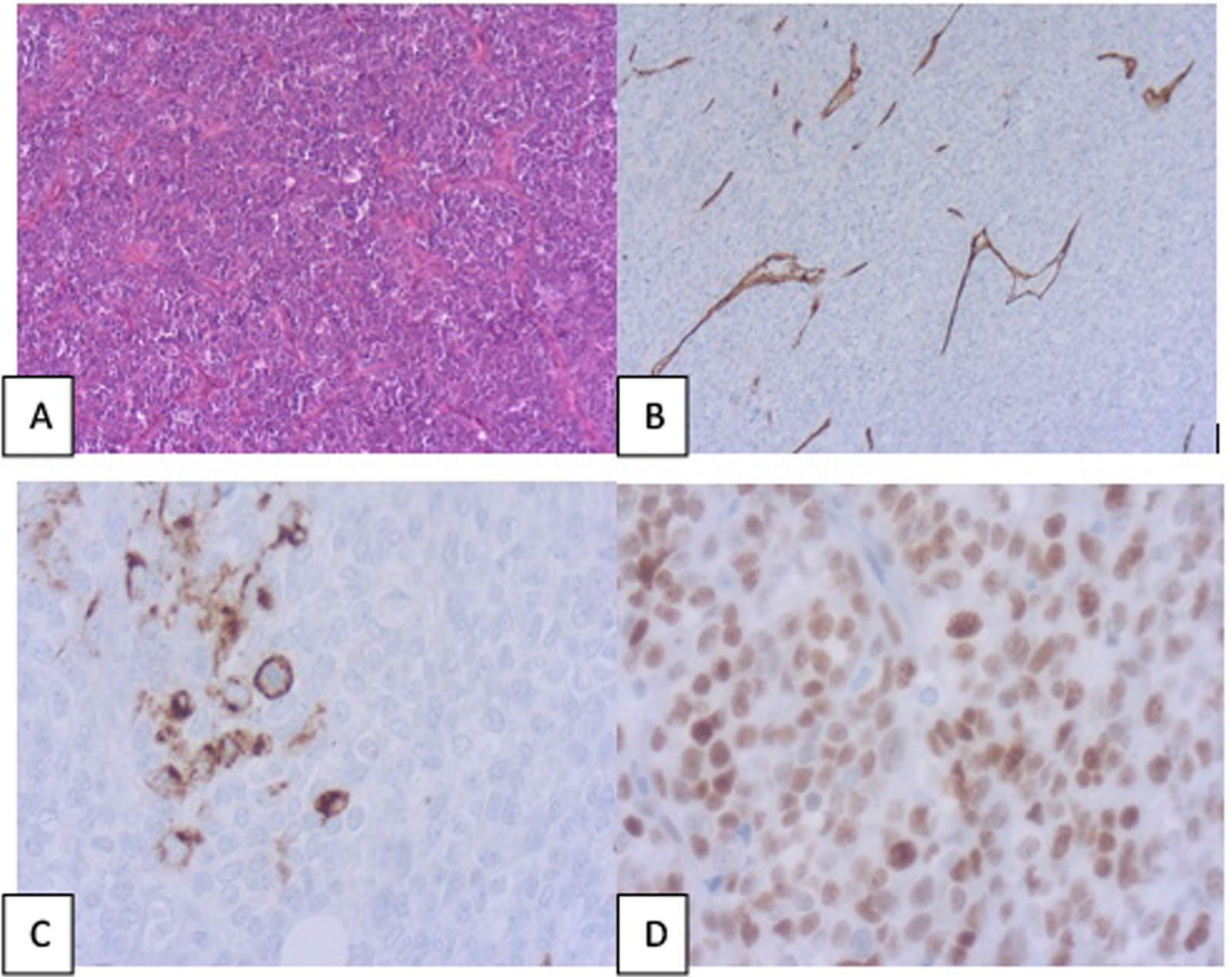
**A** Tumor proliferation made of atypical cuboidal tumor cells arranged in sheets network (HEx100); **B** CD34 staining within highlighting the staghorn vascular network; **C** positivity of scattered cells with EMA; **D** diffuse nuclear positive staining with TLE1

After 2 weeks, a laparotomy was performed with the removal of the mesenteric mass, which was locally invading the greater omentum and the appendiceal wall. The resected mass was ill-defined, non-encapsulated, sized 19 × 17 cm, and weighed 1893 g. It was firm, fleshy with a white cut surface harboring hemorrhagic and mucoid areas. The mass was partially connected to the appendiceal wall and the greater omentum. Microscopically, the tumor was highly cellular and showed ovoid to spindle cells arranged in sheets and vague fascicles with hemangiopericytoma-like areas. Tumor cells display mild nuclear atypia and the mitotic rate was 18 mitoses⁄10 HPF. Mucoid regions exhibited hemorrhagic and mucinous changes in the setting of a hyaline–fibrous background. Immunochemistry revealed positive staining of the tumor cells for TLE1 and EMA (Fig. 2).

The other antibodies performed like CD117, DOG1, STAT6, and CD34 were negative. This panel of immunochemistry (Table 1) excluded GIST, leiomyosarcoma, malignant schwannoma, and solitary fibrous tumor. The detection of the SYT–SSX fusion gene transcript by PCR was not available in our institution. Thus, based on these findings, we confirmed the diagnosis of monophasic SS. The postoperative course was uneventful and the patient was discharged after 1 week.

**Table 1:** Differential diagnoses of mesenteric synovial sarcoma with immunochemistry findings

Tumor	SS	GIST	IMT	LMS	SFT	MPNST	RMS
Antibody							
CK ⁄ EMA	+	–	–	–	–	–	–
CD117	–	+	–	–	–	–	–
DOG1	–	+	–	–	–	–	–
CD34	–	+	+ ⁄–	–	+	–	–
h-caldesmon	–	–	–	+	–	–	–
TLE1	+	–	–	–	–	–	–
Myogenin	–	–	–	–	–	–	+
SMA	+ ⁄–	+ ⁄–	+ ⁄–	+	–	–	–
S100	+ ⁄–	+ ⁄–	–	–	–	+	–
Desmin	–	–	+	+	–	–	+
ALK	–	–	+	–	–	–	–
BCL2	+	–	–	–	+	–	–

*IMT* Inflammatory myofibroblastic tumor, *LMS* Leiomyosarcoma, *SFT* Solitary fibrous tumor, *MPNST* Malignant peripheral nerve sheath tumor, *RMS* Rhabdomyosarcoma

Our patient underwent adjuvant chemotherapy based on doxorubicin with a good response and she was free from disease 9 months after diagnosis.

Unfortunately, 2 months later, unfortunately, 2 months later she developed lung metises, prompting the decision to switch to second-line chemotherapy with Ifosfamide.

Four months later, the patient was admitted to hospital with abdominal pain and a follow-up CT scan showed locoregional recurrence with multiple peritoneal lesions. the patient died a month later (16 months after diagnosis).

## Discussion

SS was first described in 1893 [3] and represented mesenchymal neoplasm accounting for 2,5–10,5% of soft tissue malignancy. It occurs at any age, but it is more common in adolescents and young adults aged between 15 and 35 years [2, 4]. SS has been reported in any part of the body [6]. Classically, it rises in the deep soft tissue near the tendon sheath or joints [7]. Other anatomic sites are described too; however, primary intra-abdominal SS remains rare and has been typically reported as single cases or part of clinical series of SS. In a study conducted by Fisher et al. [8] detailing clinical and pathological data of 11 intra-abdominal SS, retro-peritoneum appears to be the most common site. Instead, primary mesenteric location with omental involvement is extremely rare. To the best of our knowledge, only ten cases have been reported to date, in the English literature [2, 4, 5, 7–13].

The most common clinical presentation is a painless mass. However, symptoms depend on the tumor size [3]. Hemorrhagic changes and necrosis within the tumor may lead to acute pain. Huges masses can be responsible for the compression of adjacent organs with non-specific signs, such as our patient who complained of violent pelvic pain mimicking a complicated ovarian tumor.

Radiologically, SS imaging findings are not specific [4]. Computed tomography (CT) shows a hypo-dense mass, which may be slightly hyper-intense on T1 or MRI. Marked heterogeneity and enhancement are highly suggestive of SS on both CT and MRI [3]. Nevertheless, diagnostic imaging plays a significant role to assess the mass extension and defining its original relation with nearby organs. SS tends to be a slow-growing mass, well-circumscribed with a firm consistency. Multi-nodular lesions, friable with poorly defined borders, are usually characterized by rapid growth and an aggressive course [3, 4].

Histologically, there are three main subtypes: biphasic SS, monophasic SS, and poorly differentiated [3, 9]. The monophasic SS is common (up to 60% of SS) and is composed traditionally of spindle cells harboring a fascicular pattern. Biphasic SS is made of two components: mesenchymal spindle cells with epithelial components arranged in a glandular pattern. This subtype represents near to 25% of all SS. The poorly differentiated SS exhibits generally epithelioid morphology with severe nuclear atypia and a high mitotic rate. SS displays a rich vascularization with an hemangiopericytomatous pattern of vessels [3, 9]. Immunohistochemistry offers a great contribution to assessing the diagnosis of SS and ruling out differential diagnoses depending on the site. Tumor cells express Cytokeratin AE1⁄AE3 and CAM5-2 in variable proportions, according to the epithelial appearance of the tumor. A significant number of cases are positive for BCL2, CD99, vimentin, EMA, and calretinin. S100 is expressed in about 30% of SS. CD34, CD117, h-caldesmon, and SMA are negative markers. Focal and weak positivity of DOG1, such in our case can be misleading, particularly in intraabdominal SS [6, 7]. TLE1 and SS18–SSX [14] are constant positive markers and are considered a good tool for SS diagnosis, and they can replace the use of FISH for identification of SYT gene break apart. Because mesenteric SS is rare, lacking specific clinical or radiological characteristics, its diagnosis should be made after ruling out other intra-abdominal soft tissue malignancies. Such a situation is challenging on biopsy due to the overlap of histologic features [11]. Since GIST is the most frequent mesenteric mesenchymal tumor, it is usually the first differential diagnosis of SS. GIST coexpresses CD34 and C-KIT, while both are negative in SS. The table below summarizes the main intraabdominal SS differential diagnosis and immunohistochemistry expression [15].

The gold standard for SS diagnosis is molecular testing for SS18–SSX aberrations, the product of t (x;18) (p11;q11) translocation, that can be detected by FISH or RT-PCR. Meanwhile, pathologists must know that sensitivity of SS18 break-apart FISH and RT-PCR is, respectively, 83% and 94% [13].

The management of intra-abdominal SS consists of wide surgical resection of the tumor with regional lymphadenectomy [5, 13, 16]. A removal of involved adjacent organs is proposed, in case of locoregional extension. Other treatment modalities such as radiotherapy and chemotherapy are discussed, but the results are not conclusive due to the limited size of the series [16]. Unfortunately, the prognosis of intra-abdominal SS is poor. According to Fisher et al. [8], average survival rates were only 17 months. Local recurrence may happen earlier if resection is incomplete.

## Conclusion

Mesenteric SS is an extremely rare form of intraabdominal SS. Clinically, even radiological findings are not specific and presentation may be misleading. The pathologic diagnosis is based on histologic features, immunochemistry staining, and ancillary molecular techniques. However, it remains challenging in a biopsy, especially with unusual immunophenotypes. Prognosis is poor and depend essentially on quality of surgical resection.

## Data Availability

All data generated during this study are included in the published article and its Additional files.

## Abbreviations

SS: Synovial sarcoma
CT: Computed tomography
MRI: Magnetic resonance imaging
GIST: Gastrointestinal stromal tumor
